# Is there an association between glomerular hyperfiltration and coronary flow velocity reserve in patients with gestational diabetes history?

**DOI:** 10.17305/bb.2024.10940

**Published:** 2024-09-04

**Authors:** Mumtaz Takir, Ozge Telci Caklili, Fatma Betul Ozcan, Adem Atici, Mustafa Caliskan

**Affiliations:** 1 Department of Endocrinology and Metabolism, Suleyman Yalcin City Hospital, Istanbul, Türkiye; 2 Department of Endocrinology and Metabolism, Kocaeli City Hospital, Kocaeli, Türkiye; 3 Department of Cardiology, Suleyman Yalcin City Hospital, Istanbul, Türkiye

**Keywords:** Gestational diabetes, glomerular hyperfiltration (GHF), coronary flow velocity reserve (CFVR), endothelial dysfunction

## Abstract

Glomerular hyperfiltration (GHF) is an early marker of chronic kidney disease (CKD) and may predict coronary microvascular dysfunction, presenting as reduced coronary flow velocity reserve (CFVR) in patients with a history of gestational diabetes mellitus (GDM). This study aimed to assess the glomerular filtration rate (GFR) and compare CFVR in patients with a history of GDM. We screened patients referred to the Cardiology Department of Istanbul Medeniyet University for angina pectoris, excluding those with positive treadmill test results. Women with a history of GDM were categorized into three groups based on GFR levels: Group 1 (60–89 mL/min), Group 2 (90–119 mL/min), and Group 3 (≥ 120 mL/min). Coronary diastolic peak velocities were measured at baseline and after dipyridamole administration, with CFVR defined as the ratio of hyperemic to baseline diastolic peak velocities. The homeostasis model assessment of insulin resistance (HOMA-IR) and body mass index were derived from patient files. A total of 166 patients were included: 57 in Group 1, 80 in Group 2, and 29 in Group 3. HOMA-IR was higher in Group 3 compared to Group 2 (*P* < 0.05). Group 1 had the lowest CFVR (2.3 ± 0.3%), Group 2 had the highest (2.5 ± 0.3%), and Group 3 showed a moderate decrease in CFVR (2.4 ± 0.3%). Multivariate linear regression analysis revealed that HbA1c was independently associated with CFVR. In patients with GDM, GHF is associated with reduced CFVR, linked to metabolic parameters such as HbA1c and HOMA-IR. Interventions to improve metabolic health may prevent cardiovascular disease in these patients.

## Introduction

Chronic kidney disease (CKD) is associated with decreased glomerular filtration rate (GFR). However, increased GFR, also known as glomerular hyperfiltration (GHF), can be detrimental as well. The increased pressure in the glomeruli causes podocyte hypertrophy and results in the detachment of podocytes and glomerular injury [1]. The stress on all filtration units affects post-filtration structures as well [2]. There are various causes of GHF including metabolic diseases. Obesity [2], diabetes [3], hypertension [4], and metabolic syndrome [5] are all associated with GHF.

Gestational diabetes mellitus (GDM) is defined as a temporary state of increased insulin resistance due to pregnancy-related hormones [6]. Although it is considered to be temporary, its lifelong implications suggest otherwise. GDM history increases the risk of type 2 diabetes [7] and atherosclerosis [8]. Although GDM is not directly related to renal complications, it increases CKD risk approximately threefold after ten years and still remains after 30 years [9].

Coronary flow velocity reserve (CFVR) is a surrogate marker to assess coronary function. It can evaluate luminal narrowing and the severity of stenosis. Additionally, in cases with normal epicardial coronary arteries, a decrease in CFVR is a manifestation of coronary microvascular derailment. The early stages of CKD can be predictive of coronary microvascular dysfunction and present itself as reduced CFVR in patients with GDM history. In this study, we aimed to assess the GFR of patients with GDM history and compare their CFVRs accordingly.

## Materials and methods

### Subjects

Patients referred to the Cardiology Department of Istanbul Medeniyet University for angina pectoris were screened. Patients with a history of GDM and who consented to participate in the study were included. Patients who were evaluated with a treadmill test and those with positive test results were excluded. Other exclusion criteria were: active smoking, pregnancy, diabetes, chronic or acute liver disease, kidney disease, and hypertension (patients under hypertensive treatment or with blood pressure >140/90 mmHg). A history of CAD (history of myocardial infarction, coronary angioplasty, coronary bypass surgery, or coronary artery stenosis on coronary angiography), resting wall motion abnormalities, severe pulmonary disease, and contraindication to adenosine were also exclusion criteria.

After applying the inclusion and exclusion criteria, patients were asked about their GDM history. Women with a GDM history were classified into three groups according to their GFR levels: Group 1: GFR between 60–89 mL/min, Group 2: 90–119 mL/min, and Group 3 ≥ 120 mL/min. GFR was calculated using the Modification of Diet in Renal Disease (MDRD) formula: GFR ═ 175×([serum creatinine]-1.154)×([age]-0.203)×(0.742 for females)×(1.212 for African–Americans).

Patients’ current metabolic parameters, including laboratory work-up, were recorded from patient files. The homeostasis model assessment of insulin resistance (HOMA-IR) was calculated using the formula: HOMA-IR ═ (Fasting plasma insulin × Fasting plasma glucose) / 22.5 [10].

### Imaging techniques

#### Echocardiographic evaluations (coronary flow reserve and diastolic function)

All echocardiographic measurements were performed using an echocardiography platform equipped with a phased-array transducer (GE Vivid 6, GE Healthcare, Piscataway, NJ, USA) by an experienced cardiologist who was blind to clinical data. Transthoracic Doppler echocardiography (TTDE) derived CFVR was done as previously described [11], using intravenous dipyridamole infusion (0.56 mg/kg over 4 min). If the acceleration in heart rate was not enough (10% increase from the baseline), another dose of dipyridamole (0.28 mg/kg over a 2-min period) was administered. The mid-distal part of the LAD was studied using the S5-1 probe, and the LAD artery was visualized by color Doppler flow mapping guidance in the modified parasternal view. For color Doppler echocardiography, the velocity range was defined as 8.9–24.0 cm/s. Blood flow velocity was measured using pulsed-wave Doppler echocardiography, using a sample volume of 3–4 mm, placed on the color signal in the distal LAD. The ultrasound beam direction was aligned with the distal LAD flow. The angle was kept small and no correction was applied. Coronary diastolic peak velocities were measured at baseline and after dipyridamole by averaging the highest three Doppler signals for each measurement. CFVR was defined as the ratio of hyperemic to baseline diastolic peak velocities. Coronary flow reserve was defined as the ratio of hyperemic flow to basal flow and a CFR ≤2.5 was accepted as abnormal. This cut-off is preferred over the conventional threshold (i.e., < 2.0) as recent findings suggest that a cut-off value of 2.5 could better predict overall cardiovascular risk in those without obstructive ischemic heart disease [12]. The heart rate was monitored continuously during the examination, and blood pressure was recorded at baseline and during hyperemia using an automatic arm sphygmomanometer. The intraobserver–intraclass correlation coefficient for CFVR measurement was 0.946.

Conventional Doppler parameters were also measured according to a standardized examination, and the final value was an average of three cardiac beats: early (E) and late (A), diastolic transmitral flow velocity, deceleration time of E wave, average of the septal and lateral annular mitral early diastolic (e’), late diastolic (a’), and systolic (S) spectral tissue Doppler velocity, and the E/e’ ratio.

### Ethical statement

Ethics committee approval was obtained from Istanbul Medeniyet University rule number 2020/0472. Rules of Helsinki Declaration were followed throughout the study. Informed consent was obtained from each patient.

### Statistical analysis

All statistical tests were conducted using the Statistical Package for the Social Sciences 25.0 for Windows (SPSS Inc., Chicago, IL, USA). The Kolmogorov–Smirnov test was used to analyze the normality of the data. Normally distributed variables were expressed as mean ± standard deviation (SD), while non-normally distributed variables were expressed as median with interquartile range (min–max). Categorical variables are presented as percentages.

A chi-square test was used to assess differences in categorical variables between groups. The primary analysis used ANOVA to compare all reported data for parametric variables, whereas the Kruskal–Wallis test was used to compare nonparametric variables between the median values of the groups. Univariate and multivariate logistic regression analyses were used to determine independent variables in predicting CFR worsening. After performing univariate analysis, significantly obtained variables were selected for multivariate logistic regression analysis using the stepwise method. The results of univariate and multivariate regression analyses were presented as odds ratios with 95% CI. Significance was assumed at a two-sided *P* < 0.05.

## Results

A total of 166 patients were included. There were 57 patients in Group 1, 80 in Group 2, and 29 in Group 3. The demographic characteristics of the cohort are presented in Table 1. Patients in Group 3 were statistically younger than those in Groups 1 and 2 (Table 1). BMI was also statistically different among groups, with Group 1 having the lowest BMI and Group 3 the highest. There was no difference between groups in terms of lipid profile. HOMA-IR was higher in Group 3 compared to Group 2 (*P* < 0.05).

**Table 1:** The demographic and clinical data of the study population

**Parameters**	**Group-1 (*n* ═ 57)**	**Group-2 (*n* ═ 80)**	**Group-3 (*n* ═ 29)**	***P* value**
Age (years)	34.1 ± 3.8^a^	33.9 ± 4.4^e^	31.7 ± 4.1 ^a e^	0.036
BMI (kg/m^2^)	25.9 ± 2.8* ^a^	27.7 ± 4.2*	29.0 ± 6.3 ^a^	0.005
Waist circumference (cm)	84.3 ± 5.5	86.0 ± 7.4	87.0 ± 9.2	0.192
HR (per minute)	84.1 ± 7.2	74.1 ± 8.6	77.6 ± 8.6	0.413
Peak heart rate (per minute)	102.6 ± 11.6	103.7 ± 10.8	103.4 ± 11.1	0.828
SAP (mmHg)	121.3 ± 9.8	117.2 ± 11.1	117.8 ± 14.5	0.098
DAP (mmHg)	77.0 ± 5.9* ^a^	74.3 ± 7.5*	71.6 ± 11.1 ^a^	0.010
*Laboratory findings*				
Fasting glucose (mg/dL)	93.0 ± 7.0	91.7 ± 7.3	92.8 ± 9.8	0.594
HBA1c (%)	5.2 ± 0.3	5.1 ± 0.4	5.3 ± 0.4	0.143
Creatinine (mg/dL)	0.9 ± 0.1* ^a^	0.7 ± 0.1* ^e^	0.6 ± 0.1 ^a e^	<0.001
Hemoglobin (g/dL)	13.7 ± 1.5* ^a^	13.2 ± 1.3*	13.0 ± 1.1 ^a^	0.032
Sodium (mmol/L)	138.5 ± 0.7	139.2 ± 2.3	139.5 ± 1.6	0.810
Potassium (mmol/L)	4.6 ± 0.7	4.5 ± 0.3	4.4 ± 0.3	0.798
Uric acid (mg/dL)	5.0 ± 0.8	4.7 ± 0.6	4.9 ± 0.7	0.089
Albumin (g/dL)	4.3 ± 0.5	4.4 ± 0.2	4.4 ± 0.3	0.809
TC (mg/dL)	189.3 ± 26.4	185.2 ± 30.9	186.1 ± 30.1	0.730
HDL-C (mg/dL)	45.0 ± 8.7	48.4 ± 10.3	49.1 ± 9.3	0.086
LDL-C (mg/dL)	119.7 ± 21.9	110.8 ± 28.7	109.2 ± 25.2	0.092
Triglyceride (mg/dL)	119.6 ± 42.8	127.5 ± 52.3	137.3 ± 89.3	0.405
CRP (mg/L) (min–max)	1.6 (0.2–9.3)	1.7 (0.1–11.7)	2.6 (0.1–15.6)	0.774
Insulin (min–max)	12.0 (3.8–28.3)* ^a^	9.4 (2.0–23.0)*	11.7 (2.0–21.9) ^a^	0.038
HOMA-IR (min–max)	2.7 (0.9–8.3)*	2.2 (0.1–5.6)* ^e^	3.0 (0.4–7.6) ^e^	0.018

*Group-1: GFR 60–89 mL/min; Group-2: 90–119 mL/min; Group-3: GFR ≥120 mL/min*. *P < 0.05 Between Group-1 and Group-2, **^a^***P* < 0.05 between Group-1 and Group-3, **^e^***P* < 0.05 between Group-2 and Group-3 groups. BMI: Body mass index; HR: Heart rate; SAP: Systolic arterial pressure; DAP: Diastolic arterial pressure; HbA1C: Glycosylated hemoglobin; TC: Total cholesterol; LDL: Low-density lipoprotein; HDL: High-density lipoprotein; HOMA-IR: Homeostasis model assessment of insulin resistance.

### Analyses of echocardiographic measurements

Left ventricle end-diastolic diameter and systolic diameter were similar among the three groups (Table 2). There were statistically significant differences between groups in terms of interventricular septum thickness (IVST) and posterior wall thickness (PWT) (Table 2). Patients in Group 1 had higher IVST (9.2 ± 0.9 mm) than patients in Group 2 (8.8 ± 0.9 mm) and Group 3 (8.7 ± 1.7 mm) (*P* < 0.05 for both). Similarly, PWT was higher in Group 1 (8.7 ± 0.9 mm) compared to Group 2 (8.1 ± 1.3 mm) and Group 3 (7.6 ± 1.6 mm) (*P* < 0.05 for both). There was no statistical difference between Group 2 and Group 3 in terms of PWT.

**Table 2:** Echocardiographic findings of the study population

**Parameters**	**Group-1 (*n* ═ 57)**	**Group-2 (*n* ═ 80)**	**Group-3 (*n* ═ 29)**	***P* value**
CFR (%)	2.3 ± 0.3*	2.5 ± 0.3*^e^	2.4 ± 0.3 ^e^	0.003
Basal CFR (cm/s)	24.8 ± 5.4*^a^	27.2 ± 7.2*	29.3 ± 6.0 ^a^	0.009
Peak CFR (cm/s)	57.8 ± 9.5*^a^	68.4 ± 14.7*	67.6 ± 12.5 ^a^	<0.001
LVEDD (mm)	45.1 ± 4.2	45.1 ± 3.1	45.6 ± 3.3	0.821
LVESD (mm)	27.9 ± 2.5	28.8 ± 6.4	28.1 ± 2.5	0.579
IVST (mm)	9.2 ± 0.9*^a^	8.8 ± 0.9*	8.7 ± 1.7 ^a^	0.049
PWT (mm)	8.7 ± 0.9*^a^	8.1 ± 1.3*	7.6 ± 1.6 ^a^	0.001
AoD (mm)	26.7 ± 3.3	26.2 ± 4.0	25.0 ± 2.9	0.105
AoS (mm)	26.9 ± 3.2	26.7 ± 3.8	26.2 ± 3.1	0.689
LA (mm)	32.2 ± 3.9	31.9 ± 3.3	33.1 ± 2.8	0.297
LVEF (%)	65.9 ± 4.6*^a^	63.4 ± 4.6*	63.1 ± 4.8 ^a^	0.005
E (cm/s)	79.9 ± 12.8 ^a^	83.7 ± 11.0^e^	89.9 ± 12.9 ^a e^	0.002
A (cm/s)	69.6 ± 17.8	66.4 ± 12.2	66.5 ± 12.5	0.407
Mitral E/A (ratio)	1.2 ± 0.2	1.2 ± 0.2	1.2 ± 0.1	0.655
IVCT (ms)	73.9 ± 18.0	66.7 ± 14.9	63.0 ± 18.3	0.056
IVRT (ms)	100.8 ± 14.0 ^a^	99.3 ± 25.6 ^e^	89.5 ± 15.5 ^a e^	0.045
Septal E’ (cm/s)	14.5 ± 0.7	8.6 ± 1.3	8.1 ± 1.1	0.096
Septal A’ (cm/s)	9.0 ± 0.0	15.0 ± 4.1 ^e^	13.4 ± 4.4 ^a e^	0.449
IVRT septal (ms)	74.0 ± 5.6	78.6 ± 13.9	78.2 ± 15.7	0.910

*Group-1: GFR 60–89 mL/min; Group-2: 90–119 mL/min ; Group-3: GFR ≥120 mL/min*. *P < 0.05 Between Group-1 and Group-2, **^a^***P* < 0.05 between Group-1 and Group-3, **^e^***P* < 0.05 between Group-2 and Group-3 groups. CFR: Coronary flow reserve; LVEDD: Left ventricular end-diastolic diameter; LVESD: Left ventricular end-systolic diameter; IVST: Interventricular septum thickness; PWT: Posterior wall thickness; AoD: Aort diastol; AoS: Aort systol; LA: Left atrium; LVEF: Left ventricular ejection fraction; IVCT: Interventricular contraction time; IVRT: Interventricular relaxation time; ET: Ejection time.

Mitral E deceleration time was significantly higher in Group 3 (89.9 ± 12.9 m/s) than in Group 1 (79.9 ± 12.8 m/s) and Group 2 (83.7 ± 11.0) (*P* < 0.05, for both). No difference was observed in Mitral A deceleration time and Mitral E/A ratio.

### Analysis of CFR measurements

**Figure 1.**
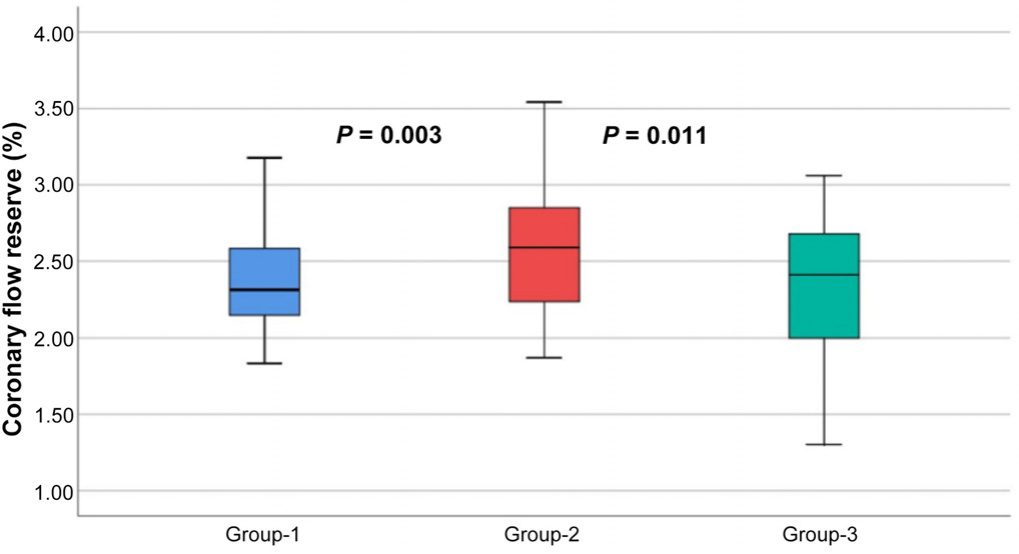
Difference in coronary flow velocity reserve according to groups.

Baseline and peak heart rates were different among the three groups (Table 2). When CFVR was assessed, patients in Group 1 had the lowest CFVR (2.3 ± 0.3%) whereas Group 2 had the highest (2.5 ± 0.3%) and Group 3 had a moderate decrease in CFVR (2.4 ± 0.3%) (*P* = 0.003 for three group comparison) (Figure 1).

### Correlation between CFVR and other variables

In univariate analysis, heart rate, HbA1c, total cholesterol, and uric acid were associated with low CFVR (Table 3). In multivariate linear regression analysis, when CFVR was taken as dependent, HbA1c was independently associated with CFVR (Table 3).

**Table 3:** Univariate and multivariate logistic regression analyzes to identify independent predictors of coronary flow velocity reserve

**Variable**	**Univariate**			**Multivariate**		
	**OR**	**95% CI**	***P***	**OR**	**95% CI**	***P***
Group-2			0.003			0.002
*Group-1*	2.26	1.13–4.53	0.001	1.45	1.17–1.98	0.001
*Group-3*	2.33	1.07–5.27	0.007	2.01	1.34–3.12	0.003
Age	1.06	0.98–1.14	0.102			
BMI	0.92	0.85–0.99	0.047	0.85	0.75–1.15	0.617
LVEF	1.08	1.01–1.16	0.014	1.06	0.96–1.15	0.205
HR	1.04	1.01–1.08	0.011	1.03	0.99–1.07	0.141
SAP	1.00	0.98–1.03	0.492			
DAP	0.99	0.95–1.03	0.728			
HbA1c	6.40	2.53–14.15	<0.001	5.11	1.83–9.22	0.002
Albumin	0.89	0.70–1.13	0.363			
TC	1.01	0.99–1.02	0.080	1.00	0.99–1.02	0.282
LDL-C	1.00	0.99–1.01	0.265			
Hemoglobin	0.94	0.75–1.18	0.633			
Uric acid	1.78	1.14–2.79	0.011	1.29	0.75–2.21	0.351
CRP	1.07	0.93–1.22	0.304	1.16	0.98–1.38	0.080

*Group-1: GFR 60–89 mL/min; Group-2: 90–119 mL/min; Group-3: GFR ≥120 mL/min*. BMI: Body mass index; LVEF: Left ventricular ejection fraction; HR: Heart rate; SAP: Systolic arterial pressure; DAP: Diastolic arterial pressure; HbA1C: Glycosylated hemoglobin; TC: Total cholesterol; LDL: Low-density lipoprotein; CRP: C-reactive protein.

## Discussion

The findings of this study highlight the complex interplay between GDM, GHF, and coronary microvascular function. Specifically, we found that patients with a history of GDM and high GFR exhibit significantly reduced CFVR. Moreover, the significant correlation between HbA1c and CFVR shows that even in patients with normal HbA1c, a GDM history can increase the risk of coronary microvascular dysfunction fivefold in the presence of GHF. To the best of our knowledge, this is the first study to test CFVR in patients with GDM and GHF and this relationship underscores the potential long-term cardiovascular risks associated with GDM emphasizing the need for early intervention and monitoring.

The significant correlation between HbA1c and CFVR indicates that glycemic control plays a crucial role in modulating cardiovascular risk in this population. Even in patients with normal HbA1c levels, a history of GDM appears to increase the risk of coronary microvascular dysfunction [13, 14]. Additionally, hyperglycemia is associated with endothelial dysfunction due to metabolic end products, such as reactive oxygen species and glycated proteins [15]. These molecules impair endothelial tissue and cause dysfunction in the microvascular circulation of the heart [16]. Our findings align with previous studies that have shown a “legacy effect” of dysglycemia, where prior episodes of high blood glucose have lasting adverse effects on vascular health [17, 18].

The pathophysiological mechanisms linking GDM, increased GFR, and reduced CFVR likely involve a combination of metabolic and hemodynamic factors. As an intricate web of vessels, the structure of glomeruli may be damaged in the presence of high glucose and later present itself as CKD [19]. Pregnancy itself can cause hyperfiltration; however, this increase in glomerular flow is temporary. GHF is one of the early signs of CKD and can appear years before CKD is established [20]. In this study, it was shown that even in the early years after birth, if GHF is present, CFVR is decreased compared to controls. This relationship is affected by HbA1c, although all patients had normal HbA1c levels. Insulin and HOMA-IR were statistically different between groups and patients with high or low GFR compared to patients with GFR between 90–119 mL/min had higher insulin and HOMA-IR. Although HOMA-IR is not a direct measurement of insulin resistance it suggests metabolic dysfunction. A large cohort study from Korea has shown that hyperfiltration is associated with metabolic disorders [21]. As glycemic control deteriorated from normal to diabetic levels, the percentage of GHF increased in the population. GHF was also associated with BMI and abdominal obesity.

CFVR is a relatively non-invasive procedure to assess the severity of luminal stenosis and coronary microvascular function. It also represents the earlier stages of microvascular dysfunction. The calculation of GFR is an easy method, even in primary care settings. As another early marker, GHF can be corrected with improvement in metabolic parameters [22]. Taken together, the presence of low CFVR and high GFR can be presumed to be a strong sign of endothelial dysfunction and appropriate measures such as intensive lifestyle changes can be taken to decrease further damage. It should also be noted that both low CFVR and high GFR are reversible. In this context, GHF should prompt healthcare professionals to facilitate better control of metabolic parameters.

The ability to predict coronary artery disease in patients with a history of GDM through simple measures like GFR assessment offers significant clinical advantages. Early identification of patients at risk can facilitate timely interventions to improve metabolic health and potentially reduce cardiovascular morbidity and mortality. Lifestyle modifications, improved glycemic control, and possibly pharmacological interventions targeting insulin resistance may benefit these patients. Future studies should aim to elucidate the precise mechanisms by which increased GFR contributes to coronary microvascular dysfunction. Longitudinal studies tracking changes in GFR, CFVR, and metabolic parameters over time would provide valuable insights into the progression of cardiovascular risk in this population. Additionally, investigating the impact of specific interventions on improving CFVR and reducing cardiovascular events in patients with GDM and increased GFR would be highly beneficial. Another important avenue for research is the potential role of genetic factors in predisposing individuals to both GDM and increased GFR. Understanding these genetic links could help identify individuals at the highest risk and lead to personalized preventive strategies.

There are some major limitations of the study. Firstly, it was conducted in a single center. A relatively low sample size in the high GFR group may also be a limitation. Secondly, the cross-sectional nature of the study limits our ability to establish causality. Longitudinal studies are necessary to determine the temporal relationship between GDM, GHF, and CFVR, and to track changes over time. Furthermore, while HOMA-IR and HbA1c were assessed, other relevant metabolic parameters, such as lipid profiles, inflammatory markers, and detailed dietary and physical activity information were not collected. These factors could provide additional insights into the metabolic health of the participants and their cardiovascular risk. Although multivariate analysis was performed, there may be residual confounding factors that were not accounted for, such as genetic predispositions, environmental factors, and the use of medications that could influence both GFR and CFVR. Addressing these limitations in future research will help to better understand the relationship between GDM, GHF, and cardiovascular risk, and to develop more effective strategies for managing these patients.

## Conclusion

In conclusion, in patients with a GDM history, the presence of GHF is associated with reduced CFVR compared to patients with normal GFR. This association is linked to metabolic parameters, such as HbA1c and insulin. Patients with GHF and GDM may benefit from interventions that promote metabolic health to prevent long-term cardiovascular disease.

## Data Availability

The data underlying this article will be shared on reasonable request to the corresponding author.
